# Retrospective analysis of renal prognosis in elderly coronary artery disease patients complicated with renal insufficiency

**DOI:** 10.18632/aging.203579

**Published:** 2021-10-04

**Authors:** Jun Li, Fa-Hu Liu, Jing Guo, Ya-Fen Yu, Chun-Qing Li

**Affiliations:** 1 Department of Nephrology, Affiliated Hospital of Jiangnan University, Wuxi 214062, Jiangsu, China; 2 Research Center, Wuxi Institute of Technology, Wuxi 214121, Jiangsu, China; 3 Department of Cardiology, Affiliated Hospital of Jiangnan University, Wuxi 214062, Jiangsu, China

**Keywords:** coronary artery disease, elderly patients, renal insufficiency, renal prognosis, retrospective analysis

## Abstract

Objective and Methods: The aim of this study was to retrospectively analyze the renal prognosis of elderly coronary artery disease (CAD) patients complicated with renal insufficiency.

Results: A total of 307 patients were included. The mean follow-up period was 25±11months. The average age was 79±7 years. In the worsening renal function group, there were higher occurrence rate of heart failure and severe coronary artery stenosis, lower rate of percutaneous coronary intervention, lower medication rate of renin-angiotensin blocker, lower plasma albumin, magnesium and hemoglobulin level. There was no significant difference in the rate of worsening renal function or gastrointestinal bleeding between patients who took anti-platelet agents/statins and those without. Patients with reduced left ventricular ejective fraction had higher rate of worsening renal function, yet lower medication rate of renin-angiotensin blockers, lower plasma albumin and hemoglobulin level. Anemia, malnutrition and worsening cardiac function were risk factors of renal function deterioration and mortality.

Conclusions: In the elderly coronary artery disease patients who had renal insufficiency, antiplatelet agents and statin have non-adverse effects on renal function; lower medication rate of renin-angiotensin blocker were found in patients with either worsening renal function or heart failure. Anemia, malnutrition and worsening cardiac function are risk factors of renal function deterioration and mortality.

## INTRODUCTION

Along with the aged trend in the country, there is an increase in the incidence of chronic kidney disease (CKD) estimated to affect more than 11% of elderly populations in China [1]. Cardiovascular disease remains the most important cause of morbidity and mortality among chronic kidney disease patients [2]. Statins are the first-line therapy to decrease low-density lipoprotein levels, which have been proved to reduce the risk of cardiovascular events [3]. Yet the data of statins in elderly coronary artery disease (CAD) patients with renal insufficiency are still uncertain.

A meta-analysis of antiplatelet therapy in chronic kidney disease patients with coronary artery disease showed a decrease in myocardial infarction and no difference in cardiovascular death, but a 1.7-fold increase in minor bleeding risk [4]. A clinical research reported that low-dose aspirin (vs.no aspirin) showed a cardiovascular benefit to Japanese diabetic patients with estimated glomerular filtration (eGFR)of 60 to 89 mL/min/1.73 m^2^. However, there is no difference of cardiovascular events or major bleeding risk among those who with lower eGFRs than 60 mL/min/1.73 m^2^ [5]. The data on antiplatelet therapy in elderly coronary artery disease patients with renal insufficiency are still unclear. Trial data showed that renin-angiotensin blocker may prevent cardiovascular events in chronic kidney disease patients [6]. It is still unknown about renal prognosis and risks of mortality in elderly coronary artery disease patients complicated with renal insufficiency. There is still uncertainty about the effect of renin-angiotensin blocker and statin on their renal function. Whether anti-platelet therapy led to the worsening renal function and gastrointestinal bleeding risks in elderly patients needs further study. This study focuses on analyzing retrospectively the data about the medication of statins, antiplatelet therapy and renin-angiotensin blocker in elderly coronary artery disease (CAD) patients complicated with renal insufficiency, the risk factors of renal prognosis and mortality.

## MATERIALS AND METHODS

### Patient selection

We conducted a retrospective study of patients who presented to the affiliated hospital of Jiangnan University between January 2016 and December 2020 with a primary diagnosis of coronary artery disease through coronary angiography. Patients were included if they were older than 65 years, presented with cardiac symptoms (chest pain, dyspnea or palpitations), received coronary angiography and were diagnosed as coronary artery disease, eGFR<60 ml/min per 1.73 m^2^ using the formulation of CKD-EPI [7]. Patients were excluded if they had concurrent liver cirrhosis, malignant tumor, severe chronic obstructive pulmonary disease, Alzheimer's disease, renal transplantation, dialysis or surgical operation within three months.

### Data collection

Both medical and medication history were recorded, including hypertension, diabetes, atrial fibrillation, left ventricular ejection fraction (LVEF) and medication of aspirin, clopidogrel, ticagrelor, statins and other drugs for cardiovascular disease. The demographics and clinical biochemical parameters were recorded at admission, including blood urea nitrogen (BUN), serum creatinine, uric acid(UA), calcium, phosphate, sodium, potassium, magnesium, liver function and lipid profile. The occurrence of anemia, gastrointestinal bleeding, double of serum creatinine and death were recorded throughout the follow-up.

### Definitions

Significant coronary artery disease was defined as one or more of the following coronary artery stenosis:①≥70% diameter stenosis; ②50% to 70% diameter stenosis with fractional flow reserve (FFR) confirmed hemodynamic significance; ③50% to 70% left main coronary artery (LMCA) disease confirmed by intravascular ultrasound (IVUS) to be significant. This definition is in keeping with The American College of Cardiology Foundation/American Heart Association Task Force on Practice Guidelines and the Society for Cardiovascular Angiography and Interventions for percutaneous coronary intervention (ACCF/AHA/SCAI) 2011 guidelines [8]. Acute myocardial infarction (includes ST-segment elevation myocardial infarction [STEMI] and non–ST-segment elevation myocardial infarction [NSTEMI]) is defined according to the Third Universal Definition of Myocardial Infarction [9]. Severe coronary artery stenosis is defined as triple-vessel coronary artery disease, that is, left anterior descending coronary artery (LAD) ≥50% diameter stenosis+ left circumflex artery (LCX) ≥50% diameter stenosis + right coronary artery (RCA) ≥50% diameter stenosis.

The definition of atrial fibrillation (AF) is in accordance with the 2014 American College of Cardiology/American Heart Association/Heart Rhythm Society (AHA/ACC/HRS) task force on practice guidelines [10].

The definition of heart failure with reduced ejection fraction (HFrEF) and heart failure with preserved ejection fraction (HFpEF). HFpEF was defined as EF ≥50%, and HFrEF was defined as LVEF <50% according to the 2016 ESC Guidelines [11, 12].

The definition of chronic kidney disease (CKD).

CKD is defined as abnormalities of kidney structure or function, presenting for more than 3 months. The stage of CKD was defined according to the estimated glomerular filtration rate (eGFR), which was calculated by formulation of CKD-EPI [7].

Hyperuricemia was defined as >6mg/dl (> 360 μmol/l) in women and >7mg/dl (>420 μmol/l) in men.

### Ethics statement

Written informed consent was obtained from each patient. This study was conducted in compliance with the Declaration of Helsinki. The study protocol was approved by the Medical Ethical Review Committee of the Affiliated Hospital of Jiangnan University.

### Statistical analysis

Continuous data are expressed as mean±standard deviation or median (interquartile range), and categorical data as percentages. Continuous data are compared among more than three groups using variance analysis and compared between two groups using Independent Sample T test. Comparisons between three groups were performed using the Kruskal–Wallis test and comparisons between two groups were performed using the Mann–Whitney U test for nonparametric data. Correlations between biochemical parameters were evaluated with a Pearson Correlation Test for continuous data and Spearman Correlation Test for nonparametric data. Continuous data before and after endpoint are analyzed using Paired Comparison. Parameters shown to correlate with death and renal deterioration were identified using a backward Binary Logistic regression analysis. All statistical analyses were performed using SPSS (SPSS Inc., Chicago, IL, USA). A p-value <0.05 was considered statistically significant.

## RESULTS

### Demographic and clinical characteristics of the 307 coronary artery disease (CAD) patients (Table 1)

**Table 1:** The medical history and baseline biochemical parameters of 307 coronary artery disease (CAD) patients with renal insufficiency.

**Medical history and medication**	**Number of cases (percentage)**	**Clinical parameter**	**Mean ± SD (median, interquartile range)**
**Medical history**	-	SBP (mmHg)	137±24
Hypertension	266(83%)	DBP (mmHg)	76±13
Diabetes	103(32%)	Albumin(g/l)	37±4
Cerebrovascular disease	134(42%)	Hemoglobulin(g/l)	120±22
Hyperuricemia	209(65%)	Platelet (per ml)	174±105
Microalbuminuria	160(50%)	LDL (mmol/l)	2.1±0.8
AMI	116(38%)	Triglyceride(mmol/l)	1.3±0.6
PCI	101(33%)	Cholesterol(mmol/l)	3.9±1.0
Atrial fibrillation	133(43.3%)	Magnesium(mmol/l)	0.8±0.2
Doubling of serum creatinine	96(31%)	Calcium(mmol/l)	2.2±0.1
**Medication history**	-	Phosphorus(mmol/l)	1.2±0.2
CCB	202(66%)	Uric acid (μmol/l)	483±135
ACEI/ARBs	98(32%)	eGFR(ml/min.1.73m^2^)	46±14
Diuretic drug	162(53%)	NT-pro BNP (pg/ml)	2848(816-8746)
Statins	251(82%)	LVEF (%)	55±10
single anti-platelet agent	123(40%)		
dual anti-platelet agent	122(40%)		
Warfarin	25(8.1%)		
Rivaroxaban or Dabigatran	8(2.6%)		
Beta-receptor blocker	168(55%)		

Note: CAD, Coronary artery disease; AMI, acute myocardial infarction; PCI, percutaneous coronary Intervention; LVEF, left ventricular ejection fraction; eGFR, estimated glomerular filtration rate; SBP, systolic blood pressure; DBP, diastolic blood pressure; CCB, calcium channel blocker; ACEI, Angiotensin-Converting Enzyme Inhibitors; ARB, Angiotensin Receptor Blockers; eGFR, estimated glomerular filtration rate; LDL, low-density lipoprotein; NT-pro BNP, Amino-terminal pro-B type natriuretic peptide.

A total of 307 patients were included. The average age was 79±7 years, the ratio of male/female is 227/80. The mean follow-up period was 25±11 months. Gastrointestinal bleeding occurred in 21cases (6.8%), doubling of serum creatinine in 96 cases (31.3%) and death in 40 cases (13.0%). The cause of death included cardiogenic shock(n=31), lethal cardiac arrhythmias(n=2), brain stem infarction(n=3), cerebral hemorrhage(n=1), severe infection(n=2) and severe gastrointestinal bleeding(n=1).

The other cardiovascular medication included Digoxin 32 (10%), Trimetazidine Dihydrochloride 201 (65%), Isosorbide Mononitrate Tablets 181 (59%), Amiodarone Hydrochloride Tablets 28 (9.1%).

### The comparison of clinical parameters between coronary artery disease (CAD) patients who had deteriorated renal function and those who had preserved renal function (Table 2)

**Table 2:** The comparison of clinical parameters between coronary artery disease (CAD) patients with deteriorated renal function and those with preserved renal function.

**Baseline characters**	**CAD patients with deteriorated renal function(n=96)**	**CAD patients with preserved renal function(n=211)**	***P***
SBP (mmHg)	137±28	137±22	*0.890*
DBP (mmHg)	76±15	75±12	*0.760*
CKMB(u/l)	26(12-44)	8(5-16)	*0.000*
cTn T(pg/ml)	421(91-930)	33(12-144)	*0.026*
cTn I(pg/ml)	3.41(0.60-16.45)	0.04(0.01-1.17)	*0.675*
Age(year)	80±8	79±6	*0.183*
eGFR(ml/min.1.73m^2^)	42±15	48±13	*0.001*
BUN (mmol/l)	11±5.0	9.3±4.6	*0.002*
UA (μmol/l)	514±153	469±124	*0.015*
Cholesterol(mmol/l)	4.0±1.0	3.8±1.0	*0.182*
LDL (mmol/l)	2.2±0.8	2.0±0.7	*0.051*
Albumin(g/l)	36±4	38±4	*0.004*
Phosphate(mmol/l)	1.2±0.3	1.1±0.2	*0.017*
Magnesium(mmol/l)	0.8±0.1	0.9±0.3	*0.019*
NT-pro BNP (pg/ml)	7471(2795-14709)	1686(499-4565)	*0.000*
LVEF	51±11	56±10	*0.000*
Hemoglobulin (g/l)	115±23	122±21	*0.017*
eGFR at endpoint (ml/min.1.73m)	33±13	54±14	*0.000*

Note: SBP, systolic blood pressure; DBP, diastolic blood pressure; CTn, cardiac troponin; eGFR, estimated glomerular filtration rate; BUN, blood urea nitrogen; UA, uric acid; LDL, low-density lipoprotein; NT-pro BNP, Amino-terminal pro-B type natriuretic peptide; LVEF, left ventricular ejection fraction.

There was higher mortality (30.2% and 5.2%, *p*<0.001), higher occurrence rate of heart failure (76.0% and 28.9%, *p*<0.001) and severe coronary artery stenosis (22.9% and 11.8%, *p*=0.013), lower rate of percutaneous coronary intervention (23.9% and 36.0%, *p*=0.036), lower medication rate of renin-angiotensin blocker (23% and 35%, *p*=0.044) in the renal function deterioration group compared with patients who had preserved renal function. As shown in Table 2, there was lower plasma albumin, magnesium, hemoglobulin level, yet higher plasma low-density lipoprotein in patients who had deteriorated renal function. Patients who had deteriorated renal function had higher serum NT-pro BNP, consistently lower left ventricular ejection fraction (LVEF) comparing with patients who had preserved renal function.

Regression analysis showed that the risk factors of renal function deterioration were lower plasma hemoglobulin level during follow-up (OR 0.921, 95%CI 0.884-0.960, *p*<0.001), lower plasma magnesium (OR 0.001, 95%CI 0.000-0.026, *p*<0.001), higher plasma low-density lipoprotein (OR 2.404, 95%CI 1.126-5.134, *p*=0.023) and worsening cardiac function (OR 9.453 95%CI 4.941-16.221, *p*<0.001), severe coronary artery stenosis(OR 2.828, 95%CI 1.877-7.799, *p*=0.004) and lower rate of percutaneous coronary intervention (OR 0.047, 95%CI 0.006-0.372, *p*=0.004).

### The comparison of clinical parameters between patients who took anti-platelet agents and those who did not take anti-platelet agent (Table 3)

**Table 3:** The comparison of clinical parameters between coronary artery disease (CAD) patients who took anti-platelet agents and those who did not take anti-platelet agent.

**Baseline characters**	**CAD patients without antiplatelet (n=60)**	**CAD patients with antiplatelet (n=247)**	***P***
SBP (mmHg)	140±28	136±23	*0.298*
DBP (mmHg)	76±13	76±13	*0.565*
CKMB(u/l)	9(5-33)	12(6-30)	*0.143*
cTn T(pg/ml)	49(12-575)	75(16-431)	*0.317*
cTn I(pg/ml)	0.15(0.01-16.00)	0.22(0.01-4.96)	*0.558*
Age(year)	80±7	79±6	*0.761*
eGFR(ml/min)	50±14	45±13	*0.011*
BUN (mmol/l)	10.0±4.7	9.8±4.8	*0.794*
Uric acid(μmol/l)	516±160	473±127	*0.067*
Cholesterol(mmol/l)	3.8±1.1	3.9±1.0	*0.719*
LDL (mmol/l)	2.1±0.8	2.0±0.8	*0.787*
Albumin(g/l)	37±4	37±4	*0.107*
Phosphate(mmol/l)	1.2±0.3	1.2±0.2	*0.483*
Magnesium(mmol/l)	0.8±0.1	0.8±0.2	*0.815*
NT-pro BNP (pg/ml)	3758(990-9253)	2576(734-7394)	*0.473*
LVEF	57±10	54±10	*0.556*
Hemoglobulin (g/l)	120±24	120±21	*0.832*
eGFR at endpoint(ml/min.1.73m^2^)	55±16	48±16	*0.027*

Note: SBP, systolic blood pressure; DBP, diastolic blood pressure; cTn, cardiac troponin; eGFR, estimated glomerular filtration rate; BUN, blood urea nitrogen; LDL, low-density lipoprotein; NT-pro BNP, Amino-terminal pro-B type natriuretic peptide; LVEF, left ventricular ejection fraction.

There were 247 patients who took anti-platelet agents, including 123 cases who took dual anti-platelet agents and 124 cases took single anti-platelet agent, and 60 patients who did not take any anti-platelet agent. The patients who took aspirin or clopidogrel showed higher medical history rate of hypertension (87% and 70%, *p*=0.004) and diabetes (36% and 13%, *p*=0.001). There was higher occurrence rate of severe coronary artery stenosis (17.8% and 5.0%, *p*=0.013) and higher rate of percutaneous coronary intervention (38.1% and 8.3%, *p*<0.001) between patients who took anti-platelet agents and those without. There was no significant difference of worsening renal function rate (29% and 40%, *p*=0.104), gastrointestinal bleeding rate (5.7% and 12%, *p*=0.099), heart failure rate (43% and 47%, *p*=0.600) or mortality (12% and 18%, *p*=0.174) between patients who took anti-platelet agents and those without. Paired Comparison also showed that there was no significant difference of eGFR before and after the medication of anti-platelet agent (t=-1.594, *p*=0.113).

There was no significant difference of worsening renal function rate (29% and 27%, *p*=0.719), gastrointestinal bleeding rate (5.7% and 5.7%, *p*=0.815), mortality (9.8 and 12.2%, *p*=0.585), hypertension (83% and 90%, *p*=0.084) or diabetes (38% and 34%, *p*=0.533) between patients who took dual anti-platelet agents and those took single anti-platelet agent. However, there was higher occurrence rate of acute myocardial infarction (62% and 22%, *p*<0.001), PCI (57% and 20%, *p*<0.001), atrial fibrillation (27% and 56%, *p*<0.001), cerebrovascular disease (37% and 52%, *p*=0.021), heart failure (50% and 35%, *p*=0.016) in patients who took dual anti-platelet agents, compared with those who took single anti-platelet agent. Patients who took dual anti-platelet agents had higher serum CK-MB and cardiac troponin T comparing with those who took single anti-platelet agent. There was no significant difference of plasma low-density lipoprotein, hemoglobulin and renal function between patients who took dual anti-platelet agents and those took single anti-platelet agent.

### The comparison of clinical parameters between patients who took oral anticoagulants and those who did not

Although there was higher occurrence rate of gastrointestinal bleeding in the patients who took warfarin compared with patients who did not take warfarin (z=-3.54, P<0.001); however, there was no significant difference of mortality and renal deterioration rate between the two groups (z=-1.079, *p*=0.281; z=-0.347, *p*=0.731, respectively). There was no significant difference of gastrointestinal bleeding rate in patients who took novel oral anticoagulants compared with patients who did not take novel oral anticoagulants (z=-0.775, *p*=0.438); And there was no significant difference of mortality and renal deterioration rate between the two groups (z=-1.108, *p*=0.268; z=-0.384, *p*=0.701, respectively).

### The comparison of clinical parameters between patients who took statins and those who took no statins (Table 4)

**Table 4:** The comparison of clinical parameters between coronary artery disease (CAD) patients who took statins and those who took no statins.

**Baseline characters**	**CAD patients without statin (n=56)**	**CAD patients with statin agent (n=251)**	***P***
SBP (mmHg)	139±27	137±23	*0.563*
DBP (mmHg)	76±14	76±13	*0.951*
CKMB(u/l)	12(6-41)	12(6-26)	*0.551*
cTn T(pg/ml)	155(21-650)	66(14-422)	*0.715*
cTn I(pg/ml)	2.40(0.01-16.00)	0.19(0.01-4.16)	*0.637*
Age(year)	80±7	79±7	*0.695*
eGFR(ml/min.1.73m^2^)	46±16	46±13	*0.930*
BUN (mmol/l)	10.6±5.2	9.7±4.7	*0.202*
Uric acid(μmol/l)	529±171	472±124	*0.026*
Cholesterol(mmol/l)	4.0±1.0	3.9±1.0	*0.779*
LDL (mmol/l)	2.2±0.7	2.0±0.8	*0.267*
Albumin(g/l)	36±4	37±4	*0.034*
Phosphate(mmol/l)	1.2±0.4	1.1±0.2	*0.075*
Magnesium(mmol/l)	0.8±0.1	0.8±0.2	*0.817*
NT-pro BNP (pg/ml)	4420(1589-10645)	2492(697-7529)	*0.177*
Hemoglobulin (g/l)	119±24	120±21	*0.640*
eGFR at endpoint(ml/min)	46±17	49±16	*0.485*

Note: SBP, systolic blood pressure; DBP, diastolic blood pressure; cTn, cardiac troponin; eGFR, estimated glomerular filtration rate; BUN, blood urea nitrogen; LDL, low-density lipoprotein; NT-pro BNP, Amino-terminal pro-B type natriuretic peptide; LVEF, left ventricular ejection fraction.

The patients who took statins had higher medical history rate of hypertension (86.0% and 71.4%, *p*=0.05), diabetes (36.2% and 12.5%, *p*=0.001), higher occurrence rate of AMI (41.0% and 23.2%, *p*=0.008), higher rate of percutaneous coronary intervention (36.6% and 12.5%, *p*=0.001) and lower serum uric acid comparing with patients who did not take statins. There was no significant difference of worsening renal function rate between patients who took statins and those without. Paired Comparison also showed that there was no significant difference of eGFR before and after medication of statins (t=-1.699, *p*=0.091).

### The comparison of clinical parameters between survivors and cardiovascular disease patients who died during follow-up

The median (interquartile range) follow-up period of the dead CAD patients is 2.0(0.5-16.2) months; and the median follow-up period of CAD survivor is 24(24-31) months. The coronary artery disease patients who died had higher occurrence rate of acute myocardial infarction (65% and 34%, *p*<0.001), higher occurrence rate of gastrointestinal bleeding (15.0% and 5.6%, *p* =0.029), worsening renal function (72% and 25%, *p*<0.001), heart failure (85.0% and 37.4%, *p* <0.001), and severe coronary artery stenosis (27.5% and 13.4%, *p*=0.022), lower medication rate of renin-angiotensin blocker (17.5% and 34%, *p*=0.036), lower rate of percutaneous transluminal coronary intervention (20.0% and 34.4%, *p*=0.033) in comparison with the living. There was no difference of antiplatelet agents (67 % and 82%, *p*=0.125) and statins (70 % and 83%, *p*=0.075) medication between the dead and the living. There was lower plasma albumin (35±4 and 37±4g/l, *p*=0.011), lower hemoglobulin (112±26 and 121±21, *p*=0.038) in coronary artery disease patients who died compared with the living.

Regression analysis showed that the risk factors of death were mainly worsening cardiac function (OR 6.349, 95%CI 1.86-21.668, *p*=0.003), deteriorated renal function (OR 6.28, 95%CI 2.18-18.085, *p*=0.001), lower rate of percutaneous coronary intervention (OR 0.220, 95%CI 0.068-0.716, *p*=0.012), and occurrence of AMI (OR 4.217, 95%CI 1.483-11.986, *p*=0.007), lower plasma hemoglobulin level during follow-up (OR 0.942, 95%CI 0.905-0.980, *p*=0.003).

### The comparison of clinical parameters among HFpEF, HFrEF and patients with normal cardiac function (Table 5)

**Table 5:** The comparison of clinical parameters among HFrEF patients, HFpEF patients and coronary artery disease patients with normal cardiac function.

**Baseline characters**	**HFrEF patients (n=89)**	**HFpEF patients (n=102)**	**Normal cardiac function (n=116)**
SBP (mmHg)	132±23	138±26	140±22**
DBP (mmHg)	74±12	77±14*	75±12
MAP (mmHg)	93±14	98±16*	97±14*
CKMB(u/l)	21(11-41)	12(6-35)	7(5-13) **
cTn T(pg/ml)	347(68-966)	88(17-494)	25(12-79) **
cTn I(pg/ml)	3.9(0.08-17.80)	0.90(0.01-15.00)	0.01(0.01-0.68)
NT-proBNP	11570(4327-20192)	4145(2738-6666) **	505(248-993) **
LVEF	40±6	59±5**	63±3**
Age(year)	79±7	80±7	78±6
eGFR(ml/min.1.73m^2^)	45±14	45±15	48±12
BUN (mmol/l)	11.2±5.4	10.5±5.4	8.4±3.1**
Uric acid (μmol/l)	522±150	488±135	446±114**
Cholesterol(mmol/l)	3.8±1.1	3.8±0.9	4.0±1.1
LDL (mmol/l)	2.1±0.8	2.0±0.7	2.1±0.8
Albumin(g/l)	36±4	37±4	38±3**
Phosphate(mmol/l)	1.2±0.3	1.2±0.3	1.1±0.2
Magnesium(mmol/l)	0.8±0.1	0.8±0.1	0.8±0.3
Hemoglobulin(g/l)	117±24	118±22	125±18*
eGFR at endpoint(ml/min.1.73m^2^)	42±18	48±16*	54±13**
Hemoglobulin at endpoint (g/l)	107±20	116±19**	125±20**

Note: compared with HFrEF group, *p<0.05, **p<0.01.

HFpEF, heart failure with preserved ejection fraction; HFrEF, heart failure with reduced ejection fraction; SBP, systolic blood pressure; DBP, diastolic blood pressure; cTn, cardiac trophion; NT-pro BNP, Amino-terminal pro-B type natriuretic peptide; LVEF, left ventricular ejection fraction; eGFR, estimated glomerular filtration rate; BUN, blood urea nitrogen; LDL, low-density lipoprotein.

Patients with left ventricular ejection fraction (LVEF)≥ 50% and <50% were defined coronary artery disease (CAD) patients with preserved LVEF(HFpEF) and CAD patients with reduced LVEF(HFrEF), respectively.

Coronary artery disease patients with reduced LVEF had higher prevalence rate of atrial fibrillation (51% and 33%, *p*=0.001), higher occurrence rate of worsening renal function (49% and 24%, *p*<0.001) and mortality (25% and 6%, *p*<0.001), and lower medication rate of renin-angiotensin blockers (21% and 35%, *p*<0.05) than those who with preserved LVEF. Coronary artery disease patients with reduced LVEF had higher occurrence rate of severe coronary artery stenosis (24.7% and 11.2%, *p*=0.019) and higher rate of percutaneous coronary intervention (38.2% and 31%, *p*=0.016) than those who with normal cardiac function. There was lower diastolic blood pressure, mean arterial pressure (MAP), lower plasma albumin, hemoglobulin and lower eGFR during follow-up period in patients with reduced LVEF. Paired Comparison also showed that there was no significant difference of eGFR before and after medication of renin-angiotensin blockers (t=-1.696 *p*=0.094).

## DISCUSSION

Chronic kidney disease is independently associated with increased cardiovascular disease. It is important to avoid the risk factors of worsening renal function and cardiac function in elderly coronary artery disease patients, who are usually complicated with renal insufficiency, to improve their renal prognosis and survival rate.

We find that there is lower plasma albumin and hemoglobulin in elderly coronary artery disease patients who had worsening renal function or cardiac function. Lower serum albumin and anemia may lead to prerenal hypoperfusion and renal ischemic-hypoxic injury, which aggravate the renal function. There is higher blood urea nitrogen (BUN), uric acid and trend of higher BUN / creatinine ratio in patients who had deteriorated renal function, which might be related with the use of diuretics, hypercatabolic state due to malnutrition, and occasionally gastrointestinal bleeding. However, excessive diuresis might lead to hyperuricemia, dehydration and prerenal hypoperfusion, meanwhile malnutrition brought about microinflammation and azotemia, all these aggravating renal dysfunctions especially in elderly coronary artery disease patients [13]. Both anemia and lower serum albumin level were also found in elderly coronary artery disease patients who had cardiac sudden death. It is vital to give necessary nutrition support, improve the anemia timely and prescribe the proper diuretics dose in elderly coronary artery disease patients with renal insufficiency. Sufficient blood oxygen content is vital for the cardiac and renal prognosis of coronary artery disease patients. Current hemoglobulin target guideline to renal anemia might not meet the requirement of blood oxygen supply for cardio-renal syndrome patients. With the clinical medication of hypoxia-inducible factor prolyl hydroxylase inhibitor (HIF-PHI)-Roxadustat, which takes advantage of natural physiology coordinated erythropoiesis and has potency of decreasing myocardial ischemia reperfusion injury, more clinical trials are demand to ascertain the ideal hemoglobulin target for cardio-renal syndrome patients [14, 15].

More intensive blood pressure control is associated with lower mortality risk among trial participants with hypertension and chronic kidney disease [16]. Although renin-angiotensin blockers might lead to transitory decrease of renal function because of decreasing the hyper-perfusion, hyper-pressure, and hyperfiltration of glomerulus, it is still recommended first-line antihypertensive drugs to improve ventricular remodeling and the prognosis of cardio-renal syndrome patients [17]. In this study, we found that there was lower medication rate of renin-angiotensin blockers in elderly coronary artery disease patients with reduced left ventricular ejection fraction (LVEF) or those with cardiac sudden death. We also found that there was no significant difference of eGFR before and after medication of renin-angiotensin blockers in elderly renal insufficiency patients. Angiotensin receptor neprilysin inhibitor (ARNI)-Sacubitril/valsartan, which could balance renal blood flow and improve glomerular filtration rate, is recommended as a replacement for renin-angiotensin blocker to further reduce the risk of heart failure hospitalization and death in ambulatory patients with reduced left ventricular ejection fraction [17, 18]. However, we should monitor the adverse effects of renin-angiotensin blocker and ARNI in the elderly coronary artery disease patients, especially when accompanying with prerenal hypoperfusion or combined medication of aldosterone receptor antagonists.

There is no significant difference in occurrence rate of renal function deterioration or gastrointestinal bleeding between patients who took dual anti-platelet agents and those took single anti-platelet agent. However, there is higher gastrointestinal bleeding rate in the dead. It indicates that gastrointestinal bleeding should be closely monitored especially in elderly patients who took the anti-platelet therapy. Besides, genetic testing for individualized anti-platelet medication might decrease gastrointestinal bleeding risk in elderly patients, meanwhile guarantee its efficacy [19].

Hyperlipidemia is a known risk factor for coronary artery disease patients with chronic kidney disease. Consistently, we found that plasma low-density lipoprotein is one of risk factors of renal deterioration in elderly coronary artery disease patients. Although statins are first-line therapy to reduce the risk of cardiovascular events, it is carefully prescribed in elderly renal insufficiency patients because of potential side effects (including myalgias, transaminitis, myopathy-related disability and cognitive impairment) [20]. A retrospective analysis by Dr. Rothschild failed to show a survival benefit attributable to statins in individuals aged 80 and older hospitalized with acute or chronic manifestations of coronary artery disease [21]. However, a meta-analysis of randomized clinical trials showed that intensive (vs less-intensive) statin therapy reduced the risk of cardiovascular events in older individuals [22]. In this study, we did not observe that statin (atorvastatin, rosuvastatin) medication had adverse effects on renal function of the elderly renal insufficiency patients. Besides, there was lower plasma uric acid levels in patients who took statins, which was consistent with the research of Giuseppe [23].

It is found that there is lower plasma magnesium in elderly coronary artery disease patients who had deteriorated renal function. Regression analysis also showed that lower plasma magnesium is a risk factor of renal function deterioration in elderly patients. Previous studies reported that magnesium deficiency is known to be associated with hypertension, insulin resistance, and endothelial dysfunction that contribute to both the progression of chronic kidney disease and coronary artery disease [24]. Magnesium supplementation might be a promising treatment to improve renal and cardiovascular prognosis in cardio-renal syndrome patients.

We also found that there is higher occurrence rate of acute myocardial infarction and severe coronary artery stenosis, yet lower rate of invasive revascularization intervention, lower medication rate of renin-angiotensin blocker in elderly coronary artery disease patients with renal insufficiency. Less percutaneous coronary intervention is also one risk of death in elderly coronary artery disease patients. Since cardiac sudden death was the leading cause of morality, the hopeful measurement to the improve the prognosis for the elderly cardio-renal syndrome patients might include necessary invasive revascularization intervention, secondary prevention including antiplatelet agents and statin, first-line anti-hypertension recommendation of renin-angiotensin blockers or angiotensin receptor neprilysin inhibitor (ARNI), and anemia improvement with Roxadustat. Meanwhile, preserved renal function could be vital to guarantee safe metabolism of medication and to avoid volume overload.

## CONCLUSIONS

In the elderly coronary artery disease patients who had renal insufficiency, antiplatelet agents and statin have no adverse effects on renal function; lower medication rate of renin-angiotensin blocker was found in patients with either worsening renal function or heart failure. Anemia, malnutrition and worsening cardiac function are risk factors of renal function deterioration and mortality. Except the necessary invasive revascularization intervention, secondary prevention with antiplatelet agents and statin, angiotensin receptor neprilysin inhibitor and Roxadustat might bring better renal and cardiac function prognosis for the elderly cardio-renal syndrome patients.

### Limitation

It is only a retrospective analysis with small sample sizes. Evidence-based medical research is needed for elderly coronary artery disease patients with renal insufficiency to improve their renal prognosis and survival rate.
